# A Rare Case of Primary Intrapulmonary Neurilemmoma Diagnosed in a 43-Year-Old Asymptomatic Man with a Well-defined Intrapulmonary Mass

**DOI:** 10.3389/fonc.2018.00011

**Published:** 2018-01-30

**Authors:** Serafeim Chrysikos, Maria Kaponi, Christina Triantafillidou, Theodoros Karampitsakos, Argyrios Tzouvelekis, Maria Anyfanti, Konstantinos Marossis, Marios Konstantinou, Rodoula Tringidou, Demosthenes Bouros, Katerina Dimakou

**Affiliations:** ^1^5th Pulmonology Department, Athens Chest Hospital “Sotiria”, Athens, Greece; ^2^6th Pulmonology Department, Athens Chest Hospital “Sotiria”, Athens, Greece; ^3^First Academic Department of Pneumonology, Athens Chest Hospital “Sotiria”, Medical School, National and Kapodistrian University of Athens, Athens, Greece; ^4^Thoracic Surgery Department, Athens Chest Hospital “Sotiria”, Athens, Greece; ^5^Pathology Department, Athens Chest Hospital “Sotiria”, Athens, Greece

**Keywords:** intrapulmonary schwannoma, neurilemmoma, positron-emission tomography, tumor, mass

## Abstract

Neurilemmoma (NL), also termed schwannoma, presents as a well-circumscribed and encapsulated mass in the human body and is almost always solitary. CT scan of a patient with NL shows a round, ovoid, or lobulated well-demarcated solid mass of soft tissue density. Primary intrathoracic neurogenic tumors location varies. However, the development of such tumors is by far more common in the costovertebral angle of the posterior mediastinum. Here, we report a rare case of a 43-year-old patient, never smoker and previously healthy, who presented with a mass adjacent to the right pulmonary hilum. This was an incidental finding on a chest X-ray after annual checkup at his workplace. The diagnosis was primary intrapulmonary NL. Primary intrapulmonary NL is an extremely rare tumor. However, based on the above, chest CT findings of a well-defined solid mass in an asymptomatic patient should raise the suspicion of NL, irrespective of the tumor localization.

## Introduction

Neurilemmoma (NL), also termed schwannoma, presents as a well-circumscribed and encapsulated mass in the human body and is almost always solitary. Primary nerve cell tumors of the lung are extremely rare, and they accounted for 0.2% of all pulmonary neoplasms (1). We herein report an interesting case of a 43-year-old patient of our clinic, who was diagnosed with NL, after investigation of a mass adjacent to the right pulmonary hilum, which is a rare location for NL development.

## Case Presentation

**Figure 1:**
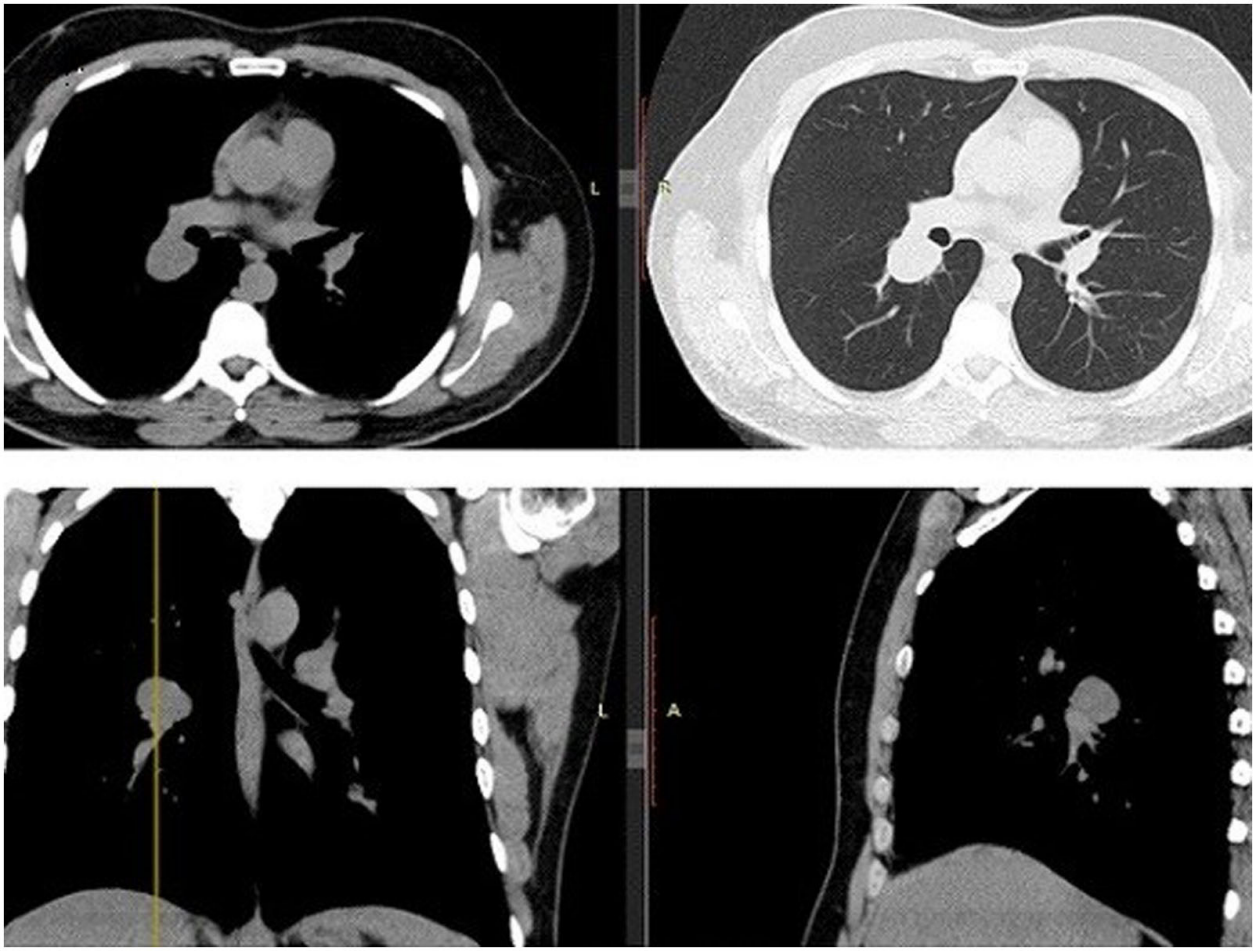
Chest CT. Transverse/coronal/sagittal planes demonstrate a round homogenous mass 32 mm × 27 mm in size in the superior segment of right lower lobe.

**Figure 2:**
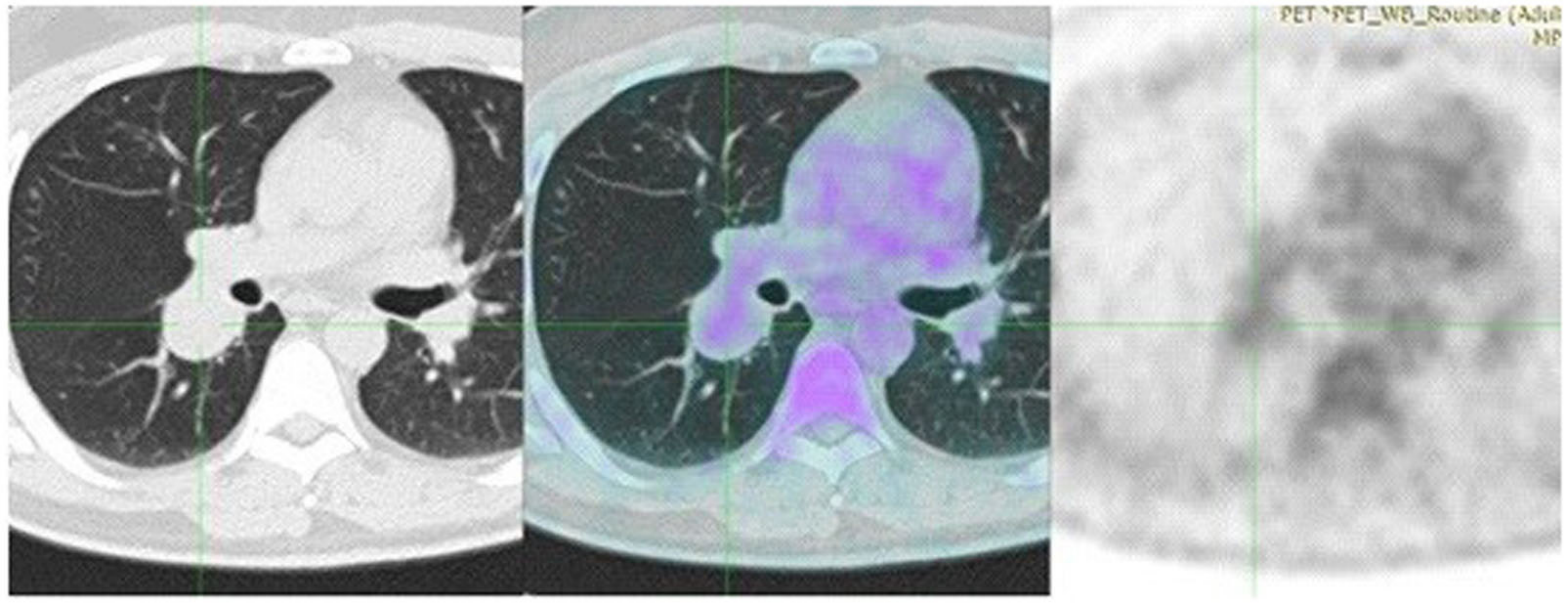
Positron-emission tomography (PET) demonstrates a diffuse, low FDG uptake (SUV_max_ 2.1) of the lesion, which exceeded that of vascular structure of the mediastinum (SUV_max_ 1.7).

A 43-year-old man, previously healthy and never smoker presented with a mass adjacent to the right pulmonary hilum, found incidentally on a chest X-ray after a checkup conducted due to a change in his position at his workplace. He was completely asymptomatic and in excellent physical condition. Typical physical examination did not reveal abnormal findings (blood pressure 120/80 mmHg, pulses 72 beats per minute, temperature 36.8°C oxygen saturation 98% on FiO_2_ 21%, lung auscultation without additional sounds). There were no palpable lymph nodes. Complete blood count and metabolic panel, urinalysis, and electrocardiography were normal. The tuberculin skin test was negative. Chest CT showed a small, well-defined, round, and solid lesion (32 mm × 27 mm), in the superior segment of right lower lobe (Figure 1). Positron-emission tomography (PET) demonstrated a diffuse, low FDG uptake (SUV_max_ 2.1) of the lesion, which exceeded that of vascular structure of the mediastinum (SUV_max_ 1.7) (Figure 2). Bronchoscopy was indicative of edema on the posterior wall of intermedius bronchus. Cytological examination from washing and brushing and endobronchial biopsy were negative for malignancy.

**Figure 3:**
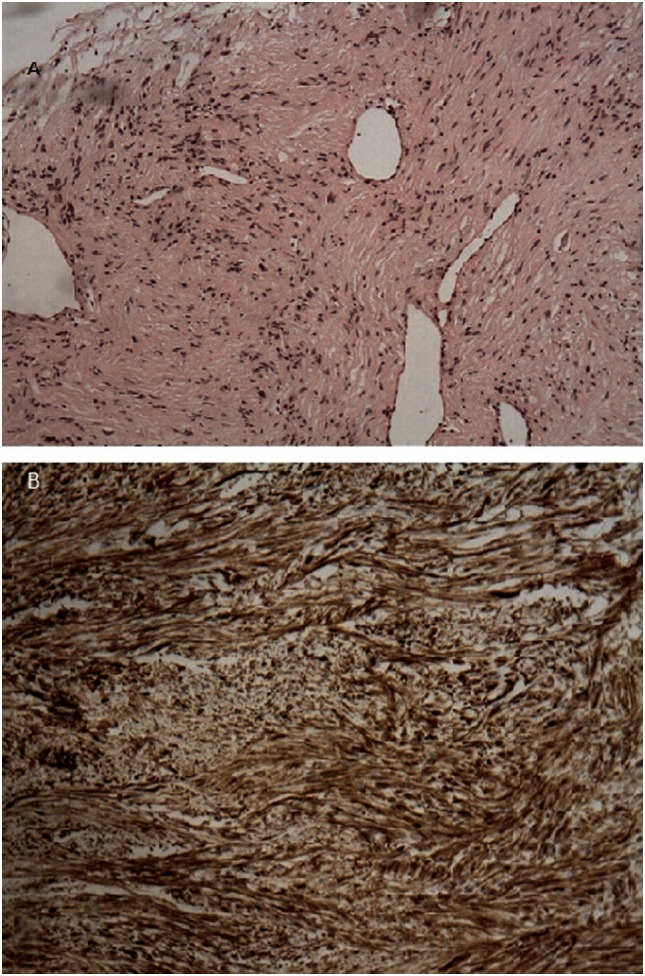
**(A)** H–E stain revealed an area of encapsulated neoplasm surrounded by lung tissue. **(B)** Microscopic examination revealed proliferation of elongated tumor cells having spindle-shaped nuclei, with cellular palisading. No necrosis or nuclear atypia was observed. Immunohistochemical staining demonstrated positive staining of the tumor cells for S-100 protein.

Given that the lesion exceeded that of vascular structure of the mediastinum, a discussion with the patient was conducted. Due to the fact that PET result was suspicious and the patient wanted a direct invasive procedure, the patient referred to a thoracic surgeon. Our working diagnosis was between a benign tumor or a low grade neuroendocrine tumor. Patient underwent right thoracotomy with right lower lobectomy and right bronchoplasty of the bronchus intermedius. Post- surgery macroscopic examination validated that tumor was well demarcated, encapsulated and round, with 3 cm diameter. There was no evidence of invasion. The color of the cut surface was yellowish. Microscopic findings included elongated tumor cells with spindle-shaped nuclei and cellular palisading. There was no evidence of nuclear atypia or necrosis. Immunohistochemical staining was positive for S-100 protein and EMA(−), PANK(−), CD68(−), SMA(−), Desmine(−), CD31(−) (Figure 3). Interestingly, the diagnosis was primary pulmonary intrapulmonary NL (schwannoma). This was an extremely interesting finding as primary intrathoracic neurogenic tumors are most frequently developed in the costovertebral angle of the posterior mediastinum. As it was expected, the 2-year follow-up revealed no further abnormalities and the patient did not report any problem with regards to his quality of life.

Every part of the procedure was approved by the Institutional Board of Chest Hospital Sotiria, Athens, Greece. Written informed consent was obtained from the participant for the publication of this case report.

## Discussion

Neurilemmoma, also termed schwannoma, presents as a well circumscribed and encapsulated mass in the human body and is almost always solitary (1). Primary intrathoracic neurogenic tumors location varies. However, the development of such tumors is by far more common in the costovertebral angle of the posterior mediastinum (2). Primary nerve cell lung tumors represent an extremely rare entity. They arise from the autonomic nerve bundles of the blood vessels and derived from the cells of Schwann’s sheath (3). Published case series of 62 patients (34 women) aged 5–83 years, with intrapulmonary or bronchial schwannomas concluded that the frequency of these tumors was 0.2% of all lung neoplasms (1, 4). Intrapulmonary schwannomas are centrally or peripherally located. Centrally located schwannomas are further subdivided into following two types: (i) intraluminal and (ii) extraluminal. Prognosis of schwannomas is good, as they represent benign, typically solitary tumors, with low rates of recurrence and malignant transformation. In addition, they are rarely multiple, and recurrence is uncommon (5, 6). Interestingly, there are reports of association between Von Recklinghausen’s disease and primary neurogenic lung tumors (2, 7).

CT scan of a patient with NL shows a round, ovoid, or lobulated well demarcated, homogeneous, solid mass with density of soft tissue. Nonetheless, NL could also present on CT as partly solid mass due to hemorrhage or necrosis. Punctuate calcifications are often noted (6).

FDG-PET/CT is generally crucial for the differentiation between malignant and benign soft tissue lesions. Data derived from retrospective studies showed that the maximum standard uptake values (SUV_max_) of NL ranged from 1.3 to 6 (mean 3.2), while malignant neurogenic tumors (MPNST) had an uptake between 4.5 and 9.7 (mean 7.0) (1, 5, 8, 9). SUV values might vary widely owing to varying degrees of microvascular density, cellularity, and vascular permeability. Areas of necrosis or cystic changes are common reasons for heterogeneous uptake (1, 5, 8, 9).

Most patients with intrapulmonary NL present with no symptoms and mass is identified incidentally on the chest X-ray, as in our case. However, a considerable percentage of patients presents with symptoms mimicking asthma, bronchitis, or poststenotic pneumonia due to airway obstruction secondary to tumor (5).

Histologically, NL is typically sharply surrounded by a fibrous capsule and is associated with two microscopic patterns—the Antoni A (cellular) and Antoni B (less cellular) (3, 4, 7). The former is associated with highly cellular areas of spindle-shaped cells interspersed with irregular, wave nuclei, and a palisade-like arrangement. The Antoni B pattern features include (i) relative hypocellularity, (ii) elongated cells with irregular fashion, (iii) separation of these cells from one another by matrix with poor or no staining for hematoxylin, eosin, and alcian blue stains. Strong, as well as diffuse staining for S-100 protein is a typical feature of these cells (3, 4, 7). Differential diagnosis of histological presence of spindle cells includes NL, leiomyoma, fibroma, angiofibroma, and sclerosing hemangioma (3, 4, 7). Therefore, immunohistochemical staining for Desmine and SMA (muscle origin tumors both smooth and skeletal), CD68 (histiocyte origin tumors), and CD31 (vascular origin tumors) is necessary (3, 4, 7).

With regards to treatment of primary intrapulmonary NL, successful surgical treatment using limited airway/sleeve resection, lobectomy, or rarely pneumonectomy for large or proximal lesions has been reported. The prognosis of intrapulmonary NL is very good, and to this end there are no reports of recurrence (1, 4, 5, 7).

## Conclusion

Primary intrapulmonary NL is an extremely rare tumor; yet, chest CT findings of a well-defined solid mass in an asymptomatic patient should raise the suspicion of NL. There is no pathognomonic radiological feature, and PET scan usually shows low uptake values; yet, variable uptake values could be shown. Due to the low malignant potential of a primary intrapulmonary NL, tumor enucleation or partial lobectomy (if possible) is sufficient unless the tumor is more proximal, which may require lobectomy. Histologic examination with positive S-100 protein immunochemistry staining is required for the diagnosis.

## Ethics Statement

This study was carried out in accordance with the recommendations of the journal, with written informed consent from the patient. The patient gave written informed consent in accordance with the Declaration of Helsinki. The protocol was approved by the Institutional Board of Chest Hospital Sotiria, Athens, Greece.

## Author Contributions

SC wrote the majority of the manuscript. TK wrote part of the manuscript and offered intellectual contribution. All the authors contributed to the results provided and approved the manuscript. AT, DB, and KD offered also intellectual contribution.

## Conflict of Interest Statement

The authors declare that the research was conducted in the absence of any commercial or financial relationships that could be construed as a potential conflict of interest.
